# Sorafenib for relapsed *FLT3*‐ITD‐positive acute myeloid leukemia postallogeneic stem cell transplantation presenting as leukemia cutis

**DOI:** 10.1002/ccr3.2487

**Published:** 2019-10-19

**Authors:** Rachel Brodie, Stephen E. Langabeer, John Quinn, Máirín E. McMenamin, Patrick J. Hayden

**Affiliations:** ^1^ Department of Haematology St. James’s Hospital Dublin Ireland; ^2^ Cancer Molecular Diagnostics St. James’s Hospital Dublin Ireland; ^3^ Department of Haematology Beaumont Hospital Dublin Ireland; ^4^ Department of Histopathology St. James’s Hospital Dublin Ireland

**Keywords:** acute myeloid leukemia, allogeneic stem cell transplantation, *FLT3* mutation, leukemia cutis, relapse, sorafenib

## Abstract

Relapse of *FLT3*‐mutated acute myeloid leukemia (AML) following allogeneic stem cell transplantation is associated with poor survival. The clinical utility of sorafenib monotherapy in this setting is described in a patient presenting as leukemia cutis.

## Graphical abstract

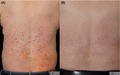

## 1 CASE

Internal tandem duplication mutations of the *FLT3* gene (*FLT3*‐ITD) are commonly acquired mutations in acute myeloid leukemia (AML) and are associated with a high risk of relapse. The FLT3 inhibitor sorafenib has been evaluated in *FLT3*‐mutated AML postallogeneic stem cell transplantation (alloSCT).^1^

**Figure 1:**
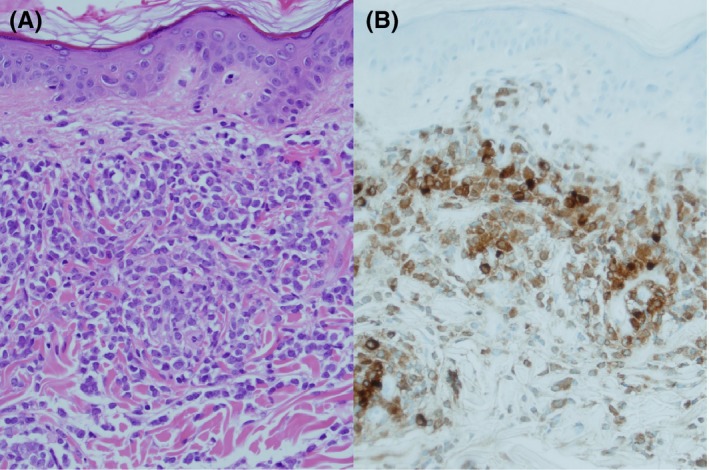
A, Skin biopsy showing dermal infiltration of myeloblasts (hematoxylin and eosin, ×200). B, Myeloblasts staining positive for myeloperoxidase (×200)

**Figure 2:**
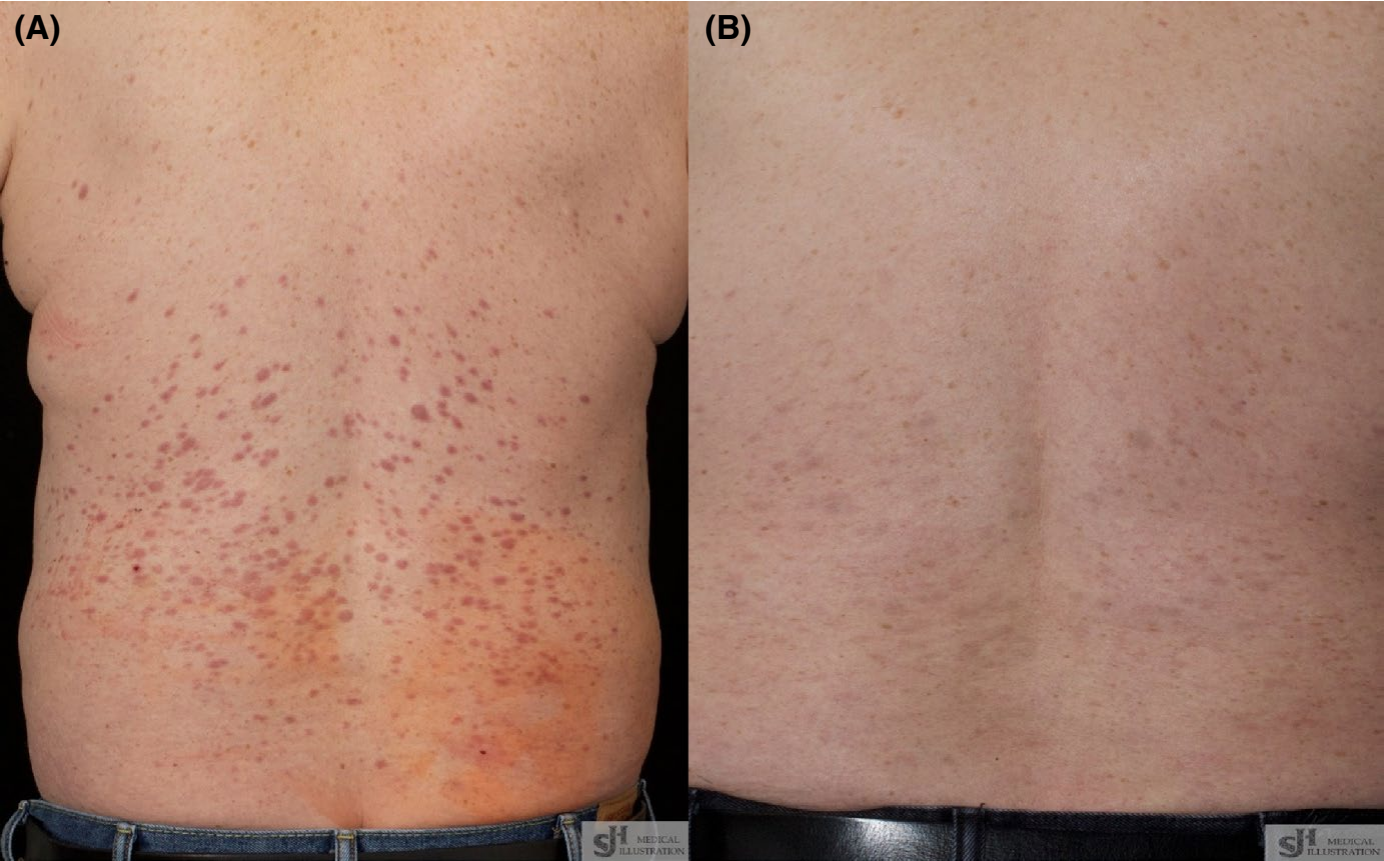
A, Presentation of leukemia cutis. B, Response after two weeks of sorafenib therapy

A 60‐year‐old man diagnosed with *FLT3*‐ITD‐positive AML in second remission underwent a sibling donor alloSCT. On Day +59, an extensive papular rash developed on his lower back, thought most likely to represent acute Graft‐Versus‐Host disease. However, skin biopsy demonstrated an infiltrate of myeloblasts consistent with leukemia cutis (Figure 1A and 1B) that was *FLT3*‐ITD mutation positive. On Day +71, he started sorafenib monotherapy (400 mg BD) resulting in a substantial clinical improvement after two weeks (Figure 2A and 2B). A donor lymphocyte infusion was administered on Day + 122 for bone marrow relapse resulting in a remission of several months until he relapsed and died on Day + 505.

Sorafenib for relapsed *FLT3*‐ITD‐positive AML presenting as leukemia cutis has rarely been described.^2^ This case highlights the need to consider leukemia cutis in the differential diagnosis of a rash early post‐alloSCT and the rapidity of the response that can be achieved with sorafenib.

## CONFLICT OF INTEREST

All authors declare no conflicts of interest.

## AUTHOR CONTRIBUTIONS

RB and SEL: collated the data. JQ and PJH: contributed to patient care and clinical information. MEM: contributed to histopathological review. All authors contributed to manuscript preparation and approved the final version.

## CONSENT

Written informed consent was obtained from the patient.

## References

[1] Antar A , Otrock ZK , El‐Cheikh J , et al. Inhibition of FLT3 in AML: a focus on sorafenib. Bone Marrow Transplant. 2017;52(3):344‐351. 27775694 10.1038/bmt.2016.251

[2] Lee SH , Paietta E , Racevskis J , Wiernik PH . Complete resolution of leukemia cutis with sorafenib in an acute myeloid leukemia patient with FLT3‐ITD mutation. Am J Hematol. 2009;84(10):701‐702. 19714594 10.1002/ajh.21511

